# Lymphoma Presenting as Acute-Onset Dysphagia

**DOI:** 10.1155/2015/745121

**Published:** 2015-11-09

**Authors:** Daniel B. Simmons, Andrew W. Bursaw

**Affiliations:** San Antonio Military Medical Center, Department of Neurology, 3551 Roger Brooke Drive, Fort Sam Houston, TX 78234, USA

## Abstract

A 61-year-old man with recent Bell's palsy developed acute vocal cord paralysis causing severe dysphagia. CSF analysis showed elevated protein and a normal cell count; contrast-enhanced MRI of the brain was normal. He was treated with IVIG for a presumed bulbar-variant AIDP and gradually improved. Six months later, the patient developed rapidly progressive hearing loss and vestibular dysfunction. Repeat MRI revealed bilateral enhancement of the eighth cranial nerves and a hypercellular mass in the left temporal lobe. Biopsy of the mass confirmed the diagnosis of diffuse large B-cell lymphoma. Lymphomatous invasion of the cranial nerves should be considered in cases of relapsing cranial neuropathies.

## 1. Introduction

In this report we describe a case of lymphoma which presented as isolated, acute-onset dysphagia. Although initially thought to be peripheral demyelinating disease of the cranial nerves, the later finding of an intracranial mass led to definitive diagnosis and treatment.

## 2. Case Presentation

A 61-year-old man presented with severe dysphagia for 3 days. He had no other neurologic complaints. His past medical history included hypertension, chronic obstructive pulmonary disease, cervical stenosis status-post anterior discectomy and fusion from C3 to C6 five months earlier, and a right-sided, idiopathic, facial paralysis four weeks earlier which resolved after administration of oral prednisone. He had a forty-pack-year tobacco history and had quit smoking four years previously. Clinical examination showed inability to swallow and a hoarse voice. The remainder of his cranial nerve exam was normal, as were his limb strength and sensation, deep tendon reflexes, and coordination testing. His gait was notable for mild difficulty with tandem walking. Computed tomography of the neck with contrast showed no evidence of a structural lesion in the pharynx. A video-fluoroscopic swallow study revealed complete true vocal fold paralysis on the right and partial paralysis on the left. A thorough rheumatologic and inflammatory laboratory workup was normal. CSF analysis revealed an elevated protein of 86 mg/dL (normal = 15–45 mg/dL) and 1 white blood cell. Pertinent infectious screening included negative cultures for cytomegalovirus, enterovirus, and herpes simplex virus; negative IgM and IgG antibodies for West Nile virus; negative reagin antibody for syphilis; and negative PCR for* Borrelia burgdorferi* DNA. MRI of the brain with gadolinium contrast was unremarkable. Nerve conduction studies including bilateral nasalis studies and blink reflexes were normal; electromyography was attempted, but the patient poorly tolerated the study and opted not to complete the evaluation.

The patient was treated with 2 gm/kg IVIG over 5 days for a presumed bulbar variant of AIDP. Antibodies to GQ1b, GD1a, GD1b, and GM-1 gangliosides were absent; paraneoplastic antibodies to Hu, Ri, and Yo antigens were negative as well. Over the course of treatment, the patient experienced no improvement in his dysphagia. A percutaneous enterogastric (PEG) tube was placed, and he was discharged to a skilled nursing facility. At follow-up one month later, his dysphagia had modestly improved, but he complained of new hearing loss, vertigo, and left-sided facial weakness. He was again treated with oral prednisone and his facial weakness resolved. His symptoms gradually improved over the next six months, a clinical course which was thought to be consistent with peripheral demyelinating disease, and he was able to resume a normal diet without the use of the PEG tube. At his next follow-up, however, the patient returned to the neurology clinic with recurrence of dysphagia, hoarseness, and markedly worsened hearing loss. At this point, his original diagnosis was in doubt, and further workup was pursued.

**Figure 1:**
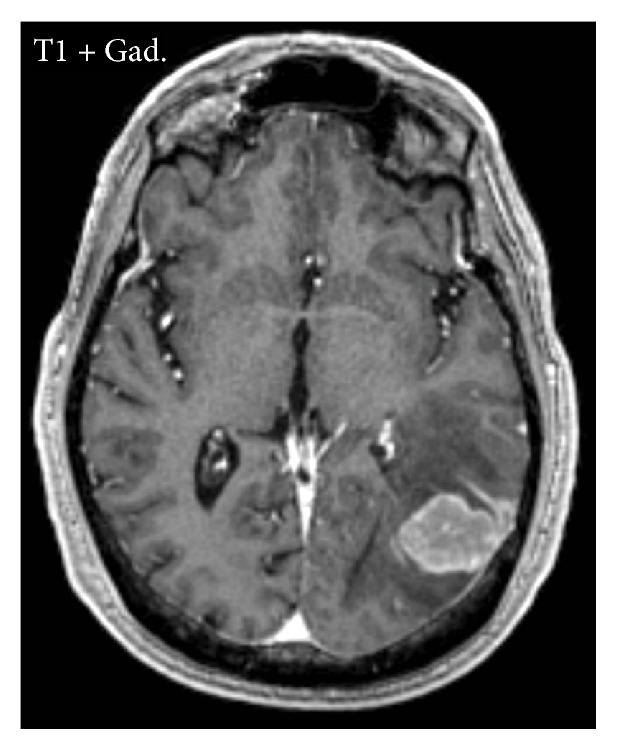
Contrast-enhanced axial T1-weighted MRI reveals a uniformly enhancing mass in the posterior temporal lobe with focal leptomeningeal enhancement.

**Figure 2:**
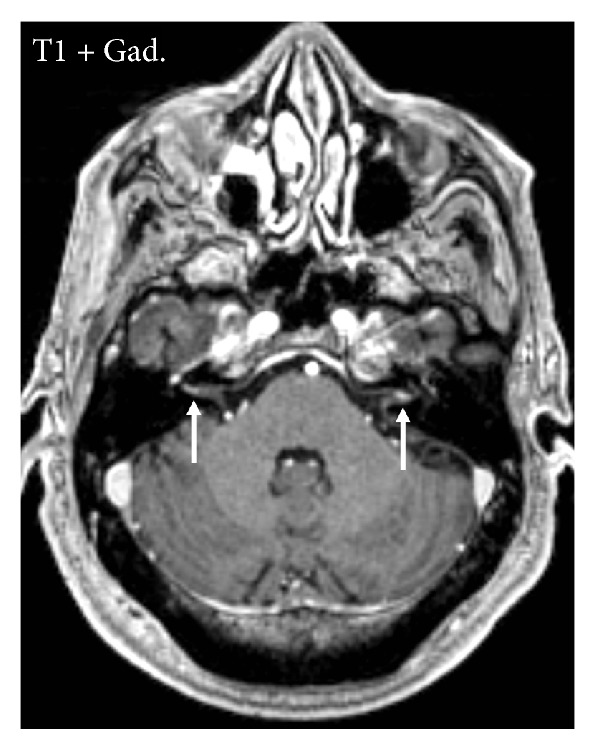
Enhancement of the eighth cranial nerves is clearly visible (arrows).

He underwent repeat MRI of the brain with gadolinium contrast. Axial T1-weighted postcontrast sequences revealed a uniformly enhancing 2 cm by 2 cm mass in the left posterior temporal lobe with surrounding vasogenic edema and focal leptomeningeal enhancement (Figure 1). Because the mass appeared to be contiguous with the dura, it was reported to be a meningioma. Additionally, there was enhancement of the bilateral eighth cranial nerves (Figure 2).

The patient was referred for biopsy. Pathologic evaluation revealed large monomorphous lymphocytes which stained positively for CD10, CD20, and BCL-6 with a proliferation index of >95%. Fluorescence in situ hybridization analysis for cMYC gene rearrangement was within normal limits. These findings were consistent with high-grade B-cell lymphoma. The final diagnosis in this case was primary neurolymphomatosis with intracranial involvement.

The patient underwent whole-body positron emission tomography (PET) and MRI of the cervical, thoracic, and lumbar spine for staging purposes. No evidence of systemic disease was found. After eight rounds of chemotherapy consisting of high-dose methotrexate, temozolomide, and rituximab, his symptoms had improved and he had no new complaints. Repeat imaging four months later showed resolution of cranial nerve enhancement and no evidence of disease recurrence.

## 3. Discussion

Neurolymphomatosis is defined by the International Primary CNS Lymphoma Collaborative Group as “nerve infiltration by neurotropic neoplastic cells in the setting of a known or unknown hematologic malignancy” [1]. The population-based incidence is unknown; however, based on a small case series, it is estimated to account for 3% of high-grade cases of non-Hodgkin's lymphoma [2]. Baehring and colleagues described four distinct phenotypes of neurolymphomatosis: painful polyneuropathy or radiculopathy (most common), cranial neuropathy, painless polyneuropathy, and mononeuropathy. Clues to the malignant nature of the neuropathy include asymmetric onset, rapid progression, or severe pain [3]. Case reports of neurolymphomatosis of the eighth cranial nerve causing hearing loss [4] and the ninth and tenth cranial nerves causing dysphagia and vocal cord paralysis [5] have been recently reported.

A high index of suspicion is required to make the diagnosis of neurolymphomatosis, especially if it is the primary presentation of malignancy. CSF cytology is positive in only 20–40% of cases [6]. Two cases of albuminocytologic dissociation on CSF studies have been previously described [7]. MRI may show peripheral nerve thickening or enhancement in 75%; ^18^FDG-PET/CT identifies a hypermetabolic focus in 84–91% of patients [8]. Nerve biopsy is considered the gold standard with definitive results in 88%, although it is performed in less than half of the cases [1].

Several different chemotherapeutic regimens have been used to treat neurolymphomatosis, most of which include high-dose intravenous methotrexate. Rituximab, which has been shown to improve survival in patients with other forms of non-Hodgkin's lymphoma, does not appear to have the same survival benefit in neurolymphomatosis [2]. Adjunctive radiation therapy is used in a minority of cases. Over half of the patients experience some degree of clinical improvement with treatment, but the effects tend to be short-lived. Median survival is approximately ten months overall, although one center reported a median survival of 21 months after excluding cases due to acute leukemia [9].

In conclusion, neurolymphomatosis is an uncommon presentation of hematologic malignancy which can progress rapidly and is difficult to diagnose. Because early identification can prevent delays in treatment and thus potentially improve survival, it is important to consider this entity in the differential diagnosis for unexplained cranial or peripheral neuropathies.
